# Supplementary material for: Global sleep homeostasis reflects temporally and spatially integrated local cortical neuronal activity
Source: eLife. 2020 Jul 2;9:e54148. doi: 10.7554/eLife.54148 (PMC7332296; doi:10.7554/eLife.54148)
Supplement: Figure 1—source data 1. [file elife-54148-fig1-data1.zip › Figure 1/Source Data Legend.pdf]

## Source Data Legend

The source data all together includes all the optimised parameters and simulated Process S time courses, and all the data required to compute them. All data time series comprise 43200 values corresponding to 48 hours sampled in 4 second epochs.

**Figure 1**

*Slow-Wave-Activity-Mouse-X.csv* includes, for mouse number X, the slow wave activity (EEG/LFP power between 0.5-4 Hz, expressed as a percentage of the average SWA in NREM sleep on the baseline day) in the frontal EEG, the mean LFP and all the individual analysed LFP channels .

*Firing-Rate-Mouse-X.*csv includes, for mouse number X, the multi-unit firing rates (spike counts in Hz) in all the individual analysed LFP channels .

*Sleep-Wake-States-X.*csv includes, for mouse number *X*, the scored sleep-wake states (either wake, NREM sleep or REM sleep) as three exhaustive and mutually exclusive binary variables, again with 4 second resolution. Alongside this is a further binary variable indicating which epochs included an artefact (and were therefore removed from analysis).

*Correlation-Values-Fig1D.csv* includes the values used to obtain the correlation coefficients presented in Figure 1D. Each row in the data table corresponds to a single analysed wake bout and the column give the mouse, LFP channel, duration of that wake bout (in seconds), mean firing rate during that wake bout in that channel (in Hz) and the change in slow wave activity from the end of the preceding NREM bout to the start of the subsequent NREM bout in that channel (in % of baseline SWA).

**Figure 3**

*Process-S-Classic-Model-SWA-Mouse-X.csv* includes, for mouse number *X*, the Process S time courses simulated with the classical state-based model (in units of SWA) in the frontal EEG, the mean LFP and all the individual analysed LFP channels.

*Process-S-FR-Model-SWA-Mouse-X.csv* includes, for mouse number *X*, the Process S time courses simulated with the firing-rate-based model (in units of SWA) in the mean LFP and all the individual analysed LFP channels.

*Parameters-Classic-Model-SWA-Mouse-X.csv* and *Process-S-FR-Model-SWA-Mouse-1.csv* include, for mouse number *X*, the associated parameter values used for these models.

**Figure 4**

*Off-Occupancy-Mouse-X.*csv includes, for mouse number X, the time course of off occupancy (in % of time window) in all the individual analysed LFP channels.

*Parameters-Off-Period-Detection.csv* includes all associated off period detection parameters (LFP amplitude threshold for slow wave detection and corresponding inter-spike interval threshold for off period acceptance) for all mice and channels.

*Interspike-Intervals-Fig4B.csv* includes all the individual values used to generate the histograms shown in Figure 4B. Each row corresponds to a detected slow wave and the two columns to the sleep-wake state in which it occurred the corresponding aligned inter-spike interval.

**Figure 5**

*Process-S-Classic-Model-Off-Mouse-X.csv* includes, for mouse number *X*, the Process S time courses measured in off occupancy and simulated with the classical state-based model (in units of off occupancy) in all the individual analysed LFP channels.

*Process-S-FR-Model-Off-Mouse-X.csv* includes, for mouse number *X*, the Process S time courses measured in off occupancy and simulated with the firing-rate-and-off-occupancy-based model (in units of off occupancy) in all the individual analysed LFP channels.

*Parameters-Classic-Model-Off-Mouse-X.csv* and *Process-S-FR-Model-Off-Mouse-1.csv* include, for mouse number *X*, the associated parameter values used for these models.
