# Supplementary material for: Global sleep homeostasis reflects temporally and spatially integrated local cortical neuronal activity
Source: eLife. 2020 Jul 2;9:e54148. doi: 10.7554/eLife.54148 (PMC7332296; doi:10.7554/eLife.54148)
Supplement: Transparent reporting form [file elife-54148-transrepform.pdf]

eLIFE
1st Floor
24 Hills Road
Cambridge CB2 1JP, UK
P 01223 855340
W elifesciences.org
T @elife

eLife's transparent reporting form

We encourage authors to provide detailed information *within their submission* to facilitate the interpretation and replication of experiments. Authors can upload supporting documentation to indicate the use of appropriate reporting guidelines for health-related research (see EQUATOR Network), life science research (see the BioSharing Information Resource), or the ARRIVE guidelines for reporting work involving animal research. Where applicable, authors should refer to any relevant reporting standards documents in this form.

If you have any questions, please consult our Journal Policies and/or contact us: editorial@elifesciences.org.

Sample-size estimation

- You should state whether an appropriate sample size was computed when the study was being designed
- You should state the statistical method of sample size computation and any required assumptions
- If no explicit power analysis was used, you should describe how you decided what sample (replicate) size (number) to use

Please outline where this information can be found within the submission (e.g., sections or figure legends), or explain why this information doesn't apply to your submission:

As stated in the Materials & Methods section the electrophysiological data used here were originally recorded for another study, which selected a sample size by comparison to existing studies within the field (McKillop et al., 2018). Since existing published Process S modelling work has also relied on similar animal numbers there was no reason to doubt that the sample would be inadequate for our purposes, which fundamentally is a proof of concept.

Replicates

- You should report how often each experiment was performed
- You should include a definition of biological versus technical replication
- The data obtained should be provided and sufficient information should be provided to indicate the number of independent biological and/or technical replicates
- If you encountered any outliers, you should describe how these were handled
- Criteria for exclusion/inclusion of data should be clearly stated
- High-throughput sequence data should be uploaded before submission, with a private link for reviewers provided (these are available from both GEO and ArrayExpress)

Please outline where this information can be found within the submission (e.g., sections or figure legends), or explain why this information doesn't apply to your submission:

This study analysed one 48-hour recording period from 6 mice. From each animal 16 LFP channels were recorded, however a total of 18 were excluded according to criteria stated in the Materials & Methods section, which were related to recording artefacts rather than physiologically relevant variability or anomaly.

Statistical reporting

- Statistical analysis methods should be described and justified
- Raw data should be presented in figures whenever informative to do so (typically when N per group is less than 10)
- For each experiment, you should identify the statistical tests used, exact values of N, definitions of center, methods of multiple test correction, and dispersion and precision measures (e.g., mean, median, SD, SEM, confidence intervals; and, for the major substantive results, a measure of effect size (e.g., Pearson's r, Cohen's d)
- Report exact p-values wherever possible alongside the summary statistics and 95% confidence intervals. These should be reported for all key questions and not only when the p-value is less than 0.05.

Please outline where this information can be found within the submission (e.g., sections or figure legends), or explain why this information doesn't apply to your submission:

Methodological details of statistical analysis are outlined in the Materials & Methods section. Whenever tests are used they are clearly described in the relevant part of the results section. Exact p-values and effect sizes are given. Boxplots include also data from individual channels/mice.

(For large datasets, or papers with a very large number of statistical tests, you may upload a single table file with tests, Ns, etc., with reference to sections in the manuscript.)

Group allocation

- Indicate how samples were allocated into experimental groups (in the case of clinical studies, please specify allocation to treatment method); if randomization was used, please also state if restricted randomization was applied
- Indicate if masking was used during group allocation, data collection and/or data analysis

Please outline where this information can be found within the submission (e.g., sections or figure legends), or explain why this information doesn't apply to your submission:

There were no experimental groups in this study. All analyses were applied to all animals.

Additional data files ("source data")

- We encourage you to upload relevant additional data files, such as numerical data that are represented as a graph in a figure, or as a summary table
- Where provided, these should be in the most useful format, and they can be uploaded as "Source data" files linked to a main figure or table
- Include model definition files including the full list of parameters used
- Include code used for data analysis (e.g., R, MatLab)
- Avoid stating that data files are "available upon request"

Please indicate the figures or tables for which source data files have been provided:

The included source data comprises all Process S time series, associated parameters and the data required to reproduce them.
Figure 1 – Slow wave activity data, multi-unit firing rate data and sleep-wake scores
Figure 3 – Parameters and Process S modelled on slow wave activity
Figure 4 – Off occupancy and off period detection parameters
Figure 5 – Parameters and Process S modelled on off occupancy
