# Supplementary material for: Global sleep homeostasis reflects temporally and spatially integrated local cortical neuronal activity
Source: eLife. 2020 Jul 2;9:e54148. doi: 10.7554/eLife.54148 (PMC7332296; doi:10.7554/eLife.54148)
Supplement: Peer review history

# Decision letter

**DOI:** 10.7554/eLife.54148.sa1

**Affiliations:** Tel Aviv UniversityIsrael

In the interests of transparency, eLife publishes the most substantive revision requests and the accompanying author responses.

**Acceptance summary:**

In many brain areas, mean firing rates of neurons are regulated by sleep-wake cycle. However, how this local homeostatic regulation is linked to a global homeostatic process determining sleep pressure remains unknown. Based on empirical and modeling work, the authors propose that global sleep homeostasis arises from a spatial and temporal integration of local activities at the level of microcircuits. This study implies that homeostatic processes, integrating the history of activity at the level of local networks, may provide intrinsic time-keeping signals that can be used to calculate global sleep need.

**Decision letter after peer review:**

[Editors’ note: the authors submitted for reconsideration following the decision after peer review. What follows is the decision letter after the first round of review.]

Thank you for submitting your work entitled "Sleep homeostasis reflects temporally integrated local cortical neuronal activity" for consideration by *eLife*. Your article has been reviewed by three peer reviewers, and the evaluation has been overseen by a Reviewing Editor and a Senior Editor.

Our decision has been reached after consultation between the reviewers. Based on these discussions and the individual reviews below, we regret to inform you that your work will not be considered further for publication in *eLife*. While all the reviewers appreciate the high quality of the data, they think that the findings are not conceptually new. The results are not qualitatively different from the established state-dependent regulation of mean firing rates and model-based predictions are not proposed.

Reviewer #1:

The paper focuses on the neuronal mechanisms that underlie sleep homeostasis. It proposes that the process which determines the need for sleep, so called process S, is not affected directly by sleep or wakefulness, but rather by deviations of local neuronal activity from a set point. The paper presents concrete mathematical models for the dynamics of process S in terms of neuronal firing rates and off periods, and shows that they could account for vigilance states and electrophysiological data collected from 6 mice over 48 hours (including a period of sleep deprivation). Thus, the paper suggests that sleep homeostasis is directly determined by intrinsic neuronal mechanisms, rather than by external cues. The proposed model for sleep homeostasis could have important implications and novel predictions for future studies on sleep regulation. Nevertheless, the fact that a model based on firing rate deviations could fit the data is not very surprising, and unexpected observations or predictions are not described, limiting the significance of the work for a wide audience. My comments relate more to the level of interpretation and predictions.

1) Although the proposed model works well, the evidence is correlational in nature rather than causal (as also mentioned in the Discussion). I would not ask for new experiments, but some non-trivial predictions regarding the effect of causal interventions should be explicitly discussed. For example, optogenetic intervention to manipulate the firing rates, even locally, is expected to affect process S in a predicted manner and provide more support for the model if the results would be consistent.

2) The paper suggests that sleep homeostasis involves multiple spatial and temporal scales. A unifying framework that could be interesting for describing this is that of critical brain dynamics, which suggests self-similarity across different spatial and temporal scales. Criticality was hypothesized in the past to in the context of sleep homeostasis [1]. Furthermore, changes in measures of criticality during sleep deprivation were described in a paper [2], on which one of the co-authors of the present paper (Achermann) is the last author. To be more specific, measures of the proximity to critical dynamics, derived from the population activity, could in principle replace the deviation of the firing rate from a set point in the proposed mathematical models. The network would be subcritical during wakefulness and supercritical during slow wave activity. One advantage of this model would be that it does not require fitting a parameter for the firing rate set point.

References:

1) Pearlmutter, B. A., and Houghton, C. J. (2009). A new hypothesis for sleep: tuning for criticality. Neural computation, 21(6), 1622-1641.

2) Meisel, C., Olbrich, E., Shriki, O., and Achermann, P. (2013). Fading signatures of critical brain dynamics during sustained wakefulness in humans. Journal of Neuroscience, 33(44), 17363-17372.

Reviewer #2:

Sleep is hypothesized to be regulated by a homeostatic sleep pressure process (and by the circadian rhythm, which is not considered in the present research). It is widely believed that this process ("process S") manifests itself in slow waves. Hence, changes in the strength of process S can be quantified by the power of slow wave activity (SWA) in segments of a few seconds in duration. This empirical behavior is traditionally approximated analytically by an exponential decay towards an asymptotic value during sleep and an inverse behavior (with a potentially different time constant and asymptote) during wakefulness. Here the authors suggest to quantify process S through the instantaneous multiunit firing rate: at any point in time where firing rate is below a certain threshold, process S decays (as during sleep) and when firing rate is above it, process S grows (as during wakefulness). A variant of the model described by an equation taking firing rates and DOWN states into consideration was also considered.

The paper shows that such quantification of process S produces trajectories that are similar to the traditional model. The question addressed by this work has a clear motivation, as firing rate level is likely more closely related to the biophysical mechanisms of process S than the EEG slow waves (for all we know EEG is an epiphenomenon). In my opinion, the result is of confirmatory nature rather than any conceptually new finding, as I do not see how any result that is qualitatively different from what is shown could have been observed, given the established finding that MUA firing rates in wakefulness are on average higher than during sleep. If the very idea that the evolution of process S can be expressed through changes in firing rate is thought to be novel and important, the paper would benefit from making this point in a concise way, using only one variant of the model.

1) I would have liked to see some form of cross-validation, e.g., by inferring the parameters separately from different portions of the data, and showing them to be in close agreement with each other. If this is not the case, the analytical model has too many parameters, which need not correspond to any biophysical processes.

2) Subsection “Data collection and pre-processing”: Some channels had an unstable MUA or had low firing rates and were excluded. The exclusion criteria seem to be subjective (no objective criteria are provided). This introduces a potential subjective bias into the findings and could preclude replication.

3) Zucca et al., 2019, demonstrate that some PV cells fire during DOWN states. This is a potential confound of defining DOWNs based on ISIs of individual channels.

4) A justification for a set point for MUA firing rate should be discussed, as different neurons can behave differently with respect to their individual set point (e.g. see Watson and Buzsaki, Neuron 2016).

Reviewer #3:

Thomas et al. present scientifically rigorous manuscript. The authors develop a novel means of modeling "process S" that is derived from the deviation of cortical firing rates from set points. The authors rigorously compare this approach to the classical model and demonstrate high agreement. The data used in this manuscript are of high spatial resolution and reveal significant differences in process S between recording channels within an animal. The work is statistically robust, and quite technical. This manuscript adds evidence to a series of papers that describe firing rate changes as a function of sleep and wake state, but makes no attempt to suggest a mechanism by which firing rates deviate or are restored. My concern with the paper is related primarily to its general interest and novelty. While the work is clearly rigorous and well executed, this study may be more appropriate for a specialized journal.

1) The findings presented in this manuscript are derived from primary motor cortex, whose activity has long been described as strongly positively related to arousal state and movement (e.g. Evarts, 1964, Sreenivasan, 2016, the Vyazovskiy group's 2016 paper by Fisher). Given evidence that, for example, visual cortical areas show less or no modulation of rate during sleep versus wake (e.g. Durkin 2016; Hengen et al. 2016), it's unclear how universalizable rules/models will be across regions. That these data and conclusions may be specific to the circuit in M1 should be made clear throughout the manuscript and explicitly addressed as a key point of interest in the Discussion.

2) One of the major speculative points in the manuscript is that the results support a model in which Process S is a time-keeping mechanism that maintains a sleep quota. The data presented seem to also be entirely consistent with a model in which waking experience-dependent alterations in neuronal physiology drive deviations in firing rates which require a homeostatic regulation of this (unidentified) physiological variable. Perhaps I have missed a key differentiator between these – if so, this should be clarified for the reader. Otherwise, the speculative model should be minimized.

3) Much of the Discussion comes across as highly speculative.

# Reply: Author response

**DOI:** 10.7554/eLife.54148.sa2

[Editors’ note: The authors appealed the original decision. What follows is the authors’ response to the first round of review.]

Our decision has been reached after consultation between the reviewers. Based on these discussions and the individual reviews below, we regret to inform you that your work will not be considered further for publication in eLife. While all the reviewers appreciate the high quality of the data, they think that the findings are not conceptually new. The results are not qualitatively different from the established state-dependent regulation of mean firing rates and model-based predictions are not proposed.

We thank the Editors for highlighting that the key message and the conceptual implications of our work were not conveyed clearly. In our revised manuscript we made an effort to redress this important omission, and here, for the benefit of the Editors and the reviewers, we would like to outline briefly the main motivation and conclusions of our study:

The ongoing discussions on the role of sleep in neural homeostasis essentially revolve around an idea that sleep and wake have opposite effects on synaptic weights or firing rates, yet the approaches used in the field are often difficult to compare directly, and studies yield inconsistent results (Aton et al., 2014; Cary and Turrigiano, 2019; Cirelli, 2017; Durkin and Aton, 2015; Frank and Cantera, 2014; Frank and Heller, 2019; Hengen et al., 2013; Hengen et al., 2016; Levenstein et al., 2017; Pacheco et al., 2019; Tononi and Cirelli, 2012, 2014, 2020). To address this deficit we used for the first time mathematical modelling inspired by the concept of sleep homeostasis (Achermann et al., 1993; Borbely, 1982; Borbely et al., 2016; Daan et al., 1984; Guillaumin et al., 2018) to investigate the dynamics of sleep-wake related firing dynamics. According to the original model, there is a mathematical relationship between duration of wakefulness or sleep and levels of “sleep need”, as reflected in EEG slow wave activity (Guillaumin et al., 2018). This view remained highly influential over more than four decades yet surprisingly it was never used before to assess the relationship between cortical firing and sleep regulation.

Our study, which combines empirical and modelling work used a step-wise approach:

– First, we estimated the parameters of the “global” homeostatic process (Process S), using a conventional EEG-based approach, where it is assumed that sleep pressure increases as a function of wake time and decreases when the animal is asleep.

– Next, we estimated the parameters of local Processes S derived from MUA and LFPs recorded in individual channels of an intra-cortically implanted microwire array, assuming that sleep pressure builds up whenever neuronal activity levels exceed a locally defined set point.

– Then, based on the knowledge that slow waves reflect local occurrence of neuronal population OFF periods, we used the latter metric to calculate a local Process S in each channel, instead of slow-wave activity derived from the EEG or LFPs.

– Finally, to bring it back to where we started, we addressed how the parameters of locally determined Processes S relate to the global dynamics of sleep pressure. Intriguingly, we observed a close correspondence between the dynamics of average Process S obtained from individual microwire recordings and the global Process S, derived from the conventional EEG recordings.

The key novel conclusion of our paper is that global sleep homeostasis is, in essence, a process arising from a spatial and temporal integration of local activities. One example of how similar work led to major advances in the field is the seminal study of Steven Reppert and Steven Strogatz (Liu et al., 1997) which concluded that "circadian period in the whole animal is determined by averaging widely dispersed periods of individual clock cells" in the suprachiasmatic nucleus – the "master clock" of the brain.

Yet another, more speculative, implication of our study is that, in contrast to common perspectives, the variables which change as a function of time spent awake or asleep might not necessarily be the ones regulated by sleep, and vice versa. Instead, Process S, by integrating “sleep-wake histories” or, more accurately, “activity histories” at local levels, provides an intrinsic time-keeping signal that precisely tracks the passage of time in each state, thereby generating a signal used to enforce that an exact quota of global sleep is obtained each day. Thus, our study offers a solution for how to bridge the gap between global and local aspects of sleep regulation.

We recognise that we did not make the rationale of our study explicit and that conceptual implications of our work did not come across clearly, which we now improved in the revised manuscript.

To this end, we made the following major changes to the manuscript:

– We significantly modified the Abstract, and expanded the Discussion to better clarify the implications of our study;

– We performed additional analyses, which explicitly illustrate that global level rate parameters are similar to the average of locally derived parameters, suggesting an integration of lower level processes by the higher level Process S (see new text in the Results section and new panels in Figure 3);

– We performed additional analysis to illustrate that the mean of all individual locally-derived Processes S closely corresponds to the global Process S derived from the EEG, in all mice (new test in Results and new Figure 6).

– We performed further validation of our approach by estimating the rate parameters separately for baseline and sleep deprivation days (new Figure 6—figure supplement 1).

– Following reviewers’ recommendations, we simplified the figures by moving analyses not essential for our key conclusions to supplementary figures (such as those previously labelled Figure 3F, G, J, and K and Figure 5G, H, K and L).

– Throughout the Results section of the manuscript, when evidence is presented which supports our key conclusions, this is more explicitly stated.

We hope these changes better support our key conclusions that local Process S reflects neuronal activity integrated over time, and that global Process S reflects local processes integrated over space.

We provide below a point by point response to the questions raised by the reviewers and indicate the changes made in the manuscript.

Reviewer #1:

[…]

1) Although the proposed model works well, the evidence is correlational in nature rather than causal (as also mentioned in the Discussion). I would not ask for new experiments, but some non-trivial predictions regarding the effect of causal interventions should be explicitly discussed. For example, optogenetic intervention to manipulate the firing rates, even locally, is expected to affect process S in a predicted manner and provide more support for the model if the results would be consistent.

We thank the reviewer for raising an important point. We acknowledge that our study is correlational, and establishing causality was beyond the scope of this specific project. Most importantly, we doubt that at this stage it is possible to design meaningful experiments to test our conclusions directly. First and foremost, this is because, as we emphasised in our manuscript, it is as yet unclear what exactly should be the variables that need to be manipulated and at what level. Equally important, the central point we wish to make is that the global dynamics of Process S arises from local activities, which should be integrated over time and space to result in the occurrence of a specific global wake-sleep pattern. Arguably, to test whether neural activity at the local level is indeed the key variable that determines the dynamics of global sleep homeostasis, it appears essential that it is recorded and manipulated simultaneously across many brain regions, and possibly this should include not only cortical but also subcortical regions. In our manuscript, we had indeed discussed previous studies where local manipulations of neural activity were performed (Finelli et al., 2000; Vyazovskiy et al., 2000; Vyazovskiy and Tobler, 2008; Vyazovskiy et al., 2004), yet these were focused on local effects only, including the example referred to by the reviewer, where optogenetic manipulation of firing rates was used (Rodriguez et al., 2016). The results of this specific study are not easy to interpret, but it provides indirect support for our conclusions. Specifically, we would predict that if Process S (particularly as measured with LFP SWA) reflects an integration of local activity-dependent processes over wider networks, the impact of local unphysiological manipulations of neural activity must be minimal because it would be “averaged out”.

To our knowledge, evidence that local cortical manipulations influence global sleep-wake architecture through mechanisms pertaining to sleep homeostasis, rather than sleep-wake state-switching, is limited if not non-existent. We argue that more widespread manipulations, consistently perturbing, for example, a whole cortical region, could be effective, and, in theory, lead to not simply local compensatory responses, but have an impact on the dynamics of global sleep-wake states. Obviously, this would not be easy to achieve methodologically, as existing approaches are often not sufficiently selective and may lead to unspecific compensatory effects.

Crucially, the relationship between local and global aspects of sleep regulation still remains not well defined. While certain inter-individual variability in global sleep amount exists, it appears to be largely conserved within species, suggesting that it must be defended against deviations (and this could be one the key functions of sleep homeostasis). On the other hand, as a result of waking activities, individual neurons or brain areas may experience widely different levels of plasticity or stress and may have very different needs for recovery (Vyazovskiy and Harris, 2013; Vyazovskiy et al., 2011). Arguably, it would be maladaptive if the global behavioural state amount and distribution has to change if only a small local network needs to benefit from global sleep. Therefore, it seems essential that global sleep must reflect an average sleep need integrated across many local brain areas. This hypothesis is consistent with the widely accepted importance of neuronal population dynamics in many brain functions, best epitomised in the idea of population coding (Panzeri et al., 2015), and also with the view of how individual oscillators in the SCN are integrated to generate a circadian rhythm of the whole animal (Achermann and Kunz, 1999; Kunz and Achermann, 2003; Liu et al., 1997). It was shown that sleep and wake duration can be influenced substantially by specific wake behaviours (Fisher et al., 2016) and by exposure to the time-free environment (Strogatz et al., 1986), suggesting an existence of underappreciated flexibility in the sleep-wake dynamics, possibly related to how efficiently sleep-wake history is integrated over time. We have included paragraphs in the Introduction and the Discussion to address this point.

2) The paper suggests that sleep homeostasis involves multiple spatial and temporal scales. A unifying framework that could be interesting for describing this is that of critical brain dynamics, which suggests self-similarity across different spatial and temporal scales. Criticality was hypothesized in the past to in the context of sleep homeostasis [1]. Furthermore, changes in measures of criticality during sleep deprivation were described in a paper [2], on which one of the co-authors of the present paper (Achermann) is the last author. To be more specific, measures of the proximity to critical dynamics, derived from the population activity, could in principle replace the deviation of the firing rate from a set point in the proposed mathematical models. The network would be subcritical during wakefulness and supercritical during slow wave activity. One advantage of this model would be that it does not require fitting a parameter for the firing rate set point.

References:

1) Pearlmutter, B. A., and Houghton, C. J. (2009). A new hypothesis for sleep: tuning for criticality. Neural computation, 21(6), 1622-1641.

2) Meisel, C., Olbrich, E., Shriki, O., and Achermann, P. (2013). Fading signatures of critical brain dynamics during sustained wakefulness in humans. Journal of Neuroscience, 33(44), 17363-17372.

We thank the reviewer for this excellent suggestion, which we indeed had considered. The notion that wakefulness may be associated with subcritical dynamics and sleep with supercritical is not incompatible with our conclusions. Still, the questions remain what are the primary variables that change as a function of sleep-wake states, and whether they represent variables that must be homeostatically regulated or they merely signal the levels of sleep need. To address these questions, we had previously attempted to characterise critical dynamics in the same dataset as used here, using a variety of approaches. This included neuronal avalanches in LFP, neuronal avalanches in multi-unit spiking, detrended fluctuation analysis and other criticality metrics (Ma et al., 2019; Meisel et al., 2013; Petermann et al., 2009; Shew et al., 2009; Wilting and Priesemann, 2018). These methods gave conflicting evidence as to the presence of sub- vs. super-criticality, and, crucially, no measures were found which consistently reflected the levels of Process S.

One possibility is that deviation from criticality might be the factor underpinning the dynamics of Process S, rather than representing a variable that reflects its instantaneous levels (Pearlmutter and Houghton, 2009). Further work is necessary to rigorously address this possibility, and crucially would require using datasets with a larger number of recording sites and well isolated individual neurons. There is a further conceptual difficulty, however, in that it is not clear how sub- vs. super-critical dynamics might be linked mechanistically to sleep homeostatic functions. Indeed, any mechanism would likely depend on firing patterns, probably changes in firing rates, since homeostasis of firing rate allows for homeostasis of criticality (Hsu and Beggs, 2006). There is the additional issue that, if indeed criticality does describe cortical dynamics, it remains unclear whether the critical (or critical-like, or critical-adjacent) state even requires homeostatic regulation, or whether it emerges intrinsically as a property of brain architecture, for example (Moretti and Munoz, 2013). By comparison, there are advantages to modelling Process S based on multi-unit activity spiking rates, instead of criticality, such as the relatively little data processing required to obtain the MUA, its relatively unambiguous physiological meaning, and well-described mechanistic association with sleep homeostasis (Vyazovskiy et al., 2009b). Indeed, mathematical models are most useful when capturing a phenomenon through the simplest possible set of mechanisms and assumptions, especially when the precise relationships between many of the relevant concepts remain unclear. Furthermore, since critical dynamics can only be measured with substantial spatial or temporal integration of activity, it would not be possible to visualise fast-scale local variability in Process S dynamics as presented here.

While there may be an important role for critical-like dynamics in the integration of Process S over many spatiotemporal scales, considerably more work would be required to explore this. Importantly, we feel that the work presented here, which relates the homeostasis of firing rates and of sleep, would represent an initial step towards such a goal, and also that the conclusions we reach here are not dependent on such an analysis being completed. In addition, once again, the point of our study is slightly different. As we emphasised, the fact that Process S increases as a function of spatial and temporal integration of local activities, does not have to be explicitly related to firing rates only, but it could be also related to other measures of neural dynamics, such as correlations at short and long temporal scales (Meisel et al., 2017; Meisel et al., 2013) or measures of complexity (Abasolo et al., 2015). We have now included a paragraph in the Discussion to address this point.

Reviewer #2:[…]

1) I would have liked to see some form of cross-validation, e.g., by inferring the parameters separately from different portions of the data, and showing them to be in close agreement with each other. If this is not the case, the analytical model has too many parameters, which need not correspond to any biophysical processes.

We thank the reviewer for an excellent suggestion, and apologise that this important aspect was not included in our analyses. We now used the opportunity to address the point of cross validation using fully automated parameter selection, which removes subjective bias, and by performing the optimisation procedure separately for baseline and sleep deprivation.

To this end, first, we applied the algorithmic optimisation approach (Matlab function *fminsearch*) on only the rate parameters (α and β) for every model we used, minimising errors individually on the baseline day or the sleep deprivation day. The other parameters (Smin, Smax, FRthresh) were left unchanged from the previously used semi-automatic approach, since algorithmic optimisation can occasionally deal poorly with high dimensionality. The resulting α and β parameters and associated model errors (median percent errors calculated over the whole 48hrs) were compared to those of the original parameter set, derived by semi-automatic selection as described in the manuscript.

We found that all parameter distributions were clustered around 100%, suggesting that parameters derived from different sections of data (baseline or sleep deprivation days) were not very different. Additionally, the distributions of error differences (compared to the errors of the original parameter set) were close to zero, suggesting that Process S modelled with parameters estimated from half of the data were equally as good fits to the data over the whole 48 hours. Importantly, the classical and firing-rate dependent models showed a very similar range of values and resulting errors, consistent with our overall conclusions. Interestingly, α was lower, and β higher, when parameter optimisation was performed for the sleep deprivation day, as compared to baseline. This held for all models, although this effect was weakest in the activity-dependent off periods model. While statistical analysis (ANOVA with factors of animal, day, model, and their interactions) supported that this effect of day was significant, the effect of the model was only significant for SWA-based Process S. Importantly, the interaction between model and day was not significant in most cases. We therefore conclude that an effect on Process S exists that is equally unaccounted for by state-based and cortical activity-based models. This may be due to factors related to behaviour or circadian phase. We now include a new figure illustrating these analyses (Figure 6—figure supplement 1), and include further discussion of these new results.

2) Subsection “Data collection and pre-processing”: Some channels had an unstable MUA or had low firing rates and were excluded. The exclusion criteria seem to be subjective (no objective criteria are provided). This introduces a potential subjective bias into the findings and could preclude replication.

We apologise for not providing a justification for excluding specific channels from our analyses, but have to emphasise that those were a minority – 18 channels across all animals out of the total 96 – which we consider an exceptional success rate for this kind of chronic electrophysiological recording performed in freely moving animals. As the reviewer likely appreciates, the methodology used for recording multiunit activity in our study, where microwire arrays were chronically implanted and MUA recorded in vivo in freely moving animals for many days, is prone to the occasional occurrence of channels which do not yield detectable neuronal activity, or result in unstable signals. To our knowledge, it is an established convention in the field that all records are visually checked and removed if deemed unreliable or unstable and this is what we had done in our previous work (Fisher et al., 2016; McKillop et al., 2018) and in the current study. The reasons for excluding a specific channel from subsequent analyses were high noise levels, an occurrence of artifacts, unsystematic rapid changes in firing rates, or major drifts in spike number across the recording period, which could not be related to the ongoing state and were therefore considered to arise from technical issues and phenomena which were not physiological.

Our approach for chronic recording of neuronal activity is based on setting an amplitude threshold for each channel whereby only those action potentials, which crossed the threshold, are saved for further analysis (Fisher et al., 2016; McKillop et al., 2018; Vyazovskiy et al., 2011; Vyazovskiy et al., 2009b). Since we record multiple animals and many channels per animal, over a number of days, it was unfortunately not feasible to record continuous raw data signal at high sampling rate in all cases. The decision on where the threshold is set is subjective, and represents a compromise between storing as many spikes as possible while preventing the intrusion of distant neuronal or system’s noise. Once the thresholds were set for a specific channel, these were never changed in the middle of an experiment, and occasionally we observed that signals deteriorated or changed in amplitude from the beginning to the end of the recording period, or that the thresholds chosen were suboptimal. We now provide further information on the recording technique used in our study in the Materials and methods section.

3) Zucca et al., 2019, demonstrate that some PV cells fire during DOWN states. This is a potential confound of defining DOWNs based on ISIs of individual channels.

This is an interesting possibility, and indeed the presence or absence of neurones which fire during population DOWN states, such as PV cells, cannot be definitively determined. It is for this reason we prefer to abstain from using the terminology of UP and DOWN state, as we record extracellular spiking activity, and in this kind of approach it remains unknown whether and which neurons are actually depolarised or hyperpolarised. The simplest possibility is that, if individual neurons were spiking when the majority of other neurons within the population are experiencing a DOWN state, we would expect that the corresponding off period would not be detectable. However, it depends strongly on the size of the recorded population, as even within a small cortical area, individual neurons may behave differently and hyperlocal off periods may occur (Fisher et al., 2016; Vyazovskiy et al., 2011). Therefore, approaches like the one used in our study are very sensitive to the precise electrode location and the amount and quality of signals recorded (see also our response above).

The off-occupancy model we used is expected to be particularly sensitive to the occurrence of neurons firing during population off periods. If neurones firing in the DOWN state contributed to the MUA, there are two possible consequences; (1) they fire so frequently that network off periods are completely undetectable in the corresponding channel, or (2) they fire only occasionally so that off periods remain detectable but may be consistently underestimated. We considered the bimodality in slow wave aligned inter-spike interval histograms to be evidence that the first case is not likely, as we observed two clear cut detectable states, corresponding to network on and off periods in all channels. If the second scenario were true, the presence of such a firing phenotype might increase the number of false negatives in off period detection, or artificially shorten the off periods which are detected, and therefore cause off occupancy to be underestimated. While this possibility cannot be entirely excluded, we surmise that this effect would be expected to be uniform across the 48hr period. Since Process S changes in proportion to off occupancy, and reflects off occupancy relative to some maximum and minimal levels, this should not present a problem for estimating Process S dynamics. If off periods are severely underestimated, this would be compensated by larger values for the beta decay rate parameter. However, the distribution of values for beta did not show abnormally high variance, which is further evidence that such an effect was minimal. Importantly, since this issue applies to all conditions (models) equally, we do not think it represents a crucial confound. It is also worth noting that previous methods for detecting off periods do so by pooling spiking over many channels (McKillop et al., 2018; Vyazovskiy et al., 2014; Vyazovskiy et al., 2011; Vyazovskiy et al., 2009b). In the case where DOWN spiking PV cells are present, this method would make the problem of false negatives even worse, since pooling spiking over channels only makes it more likely that such cells would be included, and therefore render off periods undetectable. Detecting off periods in single channels, as we did here, if anything reduces the effect of this spiking phenotype. The fact that off occupancy is strongly correlated with SWA, and that the off occupancy based model was particularly successful, support that off periods we detected are physiologically meaningful. Having said that, we, of course, cannot agree more that the possibility of occurrence of neurons firing during DOWN states, represents an important aspect that needs to be considered in future studies based on extracellular recordings of MUA and where off periods detection is performed. We address this point in the Materials and methods section and in the Discussion.

4) A justification for a set point for MUA firing rate should be discussed, as different neurons can behave differently with respect to their individual set point (e.g. see Watson and Buzsaki, 2016).

We absolutely agree with the reviewer, and had considered the possibility of estimating set points for individual neurons. In our earlier work we indeed observed individual channels and neurons behaving completely differently depending on ongoing behaviour in freely moving animals, for example some neurons increased firing rates during wheel running and others instead were virtually silent (Fisher et al., 2016). It was clear, however, that even in those cases where single units were observed in the raw data, their activity was generally not independent from concurrent MUA, which can be expected from extensive interconnectivity within local networks (Okun et al., 2015). The possibility still remains that the set point can be identified, using our approach, at the level of individual neurons, but to this end, a different approach for neural activity recording (e.g. using tetrodes), and a robust and reliable approach for spike sorting would be necessary.

The more pertinent question the reviewer is probably alluding to is how fine the spatial resolution should be to achieve meaningful conclusions about local regulation of Process S, and we do not have an answer to this question at the moment. Arguably, activities recorded locally, such as in our study, are not local, strictly speaking, as they are likely influenced by inputs arising elsewhere, and by sampling a small and randomly selected population of neurons we remain effectively blind to the activities across the rest of the brain. This is a problem that is not specific to our study and is widely acknowledged in the field. In our opinion, the next key challenges that need to be addressed are what are the biological substrates of “local” Process S, and what is the physiological relevance of its local and global dynamics. At the molecular and cellular levels, signals reflecting sleep-wake or activity-dependent changes in sleep need, likely shared between mammals and other animals, may represent changes in the availability of metabolic substrates (Krueger et al., 2008; Scharf et al., 2008), synaptic phosphoproteome (Bruning et al., 2019; Noya et al., 2019), endoplasmic reticulum stress (Williams and Naidoo, 2020), neuronal excitability (Bridi et al., 2019), synaptic strength (Tononi and Cirelli, 2014), or redox homeostasis (Kempf et al., 2019). It is likely that these changes, when they occur at the local level, may have wide-reaching effects across larger neuronal networks, which, in turn, could influence local firing activities. Thus, there is an intriguing possibility that the set point is not necessarily a fixed, immutable property, but may be by itself subject to regulation.

On a more practical point, LFP SWA is a measure derived from many neurones and population periods, while DOWN states at a single unit level are likely difficult to detect given generally low firing rates in the neocortex (McKillop et al., 2018; Watson et al., 2016). Generally, sleep homeostasis reflects slow-wave activity, which arises from population slow oscillation, which is, by definition, a network phenomenon (Neske, 2016). Therefore, we felt that for the purpose of the current study, deriving the set point for local populations (i.e. MUA from individual channels), rather than for individual neurons, is an optimal approach. We include these considerations in the Materials and methods and Discussion.

Reviewer #3:

[…]

1) The findings presented in this manuscript are derived from primary motor cortex, whose activity has long been described as strongly positively related to arousal state and movement (e.g. Evarts, 1964, Sreenivasan, 2016, the Vyazovskiy group's 2016 paper by Fisher). Given evidence that, for example, visual cortical areas show less or no modulation of rate during sleep versus wake (e.g. Durkin, 2016; Hengen et al., 2016), it's unclear how universalizable rules/models will be across regions. That these data and conclusions may be specific to the circuit in M1 should be made clear throughout the manuscript and explicitly addressed as a key point of interest in the Discussion.

We thank the reviewer for raising this point which we should have discussed in our submitted manuscript. Given the well-known and widely acknowledged differences in the anatomy, cellular composition, connectivity and function across cortical areas, we cannot agree more that the extent to which a specific result is generalizable across the cortex will always remain an important issue. As initially we did not know what to expect when we started this project, the data set recorded from the frontal motor cortex was an ideal choice, as this is a brain area where cortical activity is characteristically different between waking and sleep, and where the homeostatic response to sleep deprivation is well characterised (Fisher et al., 2016; McKillop et al., 2018; Vyazovskiy et al., 2006; Vyazovskiy and Tobler, 2005). As we discussed above, in our response to reviewers 1 and 2, the key challenge remaining at this point, is what exactly is meant by “local” and “global”, specifically when we talk about sleep regulation. We felt that our next immediate priority was not to repeat the same analyses for other cortical areas (which we are planning to do in any case), but to develop further experimental approaches that would allow us to obtain better insights into the biological substrates underlying the effects we observed. To this end, as mentioned above, we are now working on several mouse models, which present brain-wide or cortical deficits in synaptic transmission (Ang et al., 2018; Banks et al., 2020; Krone et al., 2020). Moreover, even within the frontal motor cortex, individual neurons and channels can vary hugely as a function of ongoing behaviour and activities (Fisher et al., 2016). Therefore, potential differences we may observe with other regions may not be immediately informative.

Having said that, we followed the reviewer’s advice and applied our approach to another data set that we recently recorded in the prefrontal cortex. It is clear from this example (Author response image 1) that the key observations we made for the frontal motor cortex are consistent with the dynamics observed in the prefrontal area. Specifically, our analyses suggest that in the mouse medial prefrontal cortex, the firing-rate dependent model fits SWA-derived Process S with comparable accuracy as we found in the motor cortex data. We find this preliminary analysis promising but not entirely unexpected, and further work is needed, specifically when new data will be available where the same animal is implanted in more than one cortical region.

Although relatively few cortical areas have previously been investigated systematically across the spontaneous sleep-wake cycle in adult animals, an absence of sleep-wake changes in firing in the primary visual cortex is likely to be an exception, as this phenomenon has been documented in other brain regions (Fisher et al., 2016; McKillop et al., 2018; Niethard et al., 2016; Vyazovskiy et al., 2009b). We indeed know that the homeostatic increase in SWA is lower in the posterior areas of the neocortex in rodents (Huber et al., 2000; Leemburg et al., 2010; Vyazovskiy and Tobler, 2005; Vyazovskiy et al., 2004), whereas frontal cortical areas may have an important role in slow wave generation and propagation (Vyazovskiy et al., 2009a). Importantly, we would like to emphasise that, for the position that we argue to hold, not all neurones necessarily need to have higher firing rates in wake. It was shown, for example, that neurones which increase their firing rate in NREM sleep typically have the lowest waking firing rates (Watson et al., 2016). These may therefore experience a weaker homeostatic challenge during wakefulness (such as due to cellular metabolic stress or synaptic imbalance) compared with the majority of neurones whose firing is increased by wake. These neurones might therefore express Process S (time in down states) such that it is not reflecting their own “sleep need” per se, but rather follows the need of the network in which they are embedded; hence we suggest that Process S involves an integration across space (individual neurones and networks) as well as time (individual and population firing rate history). We now discuss these points in the manuscript.

2) One of the major speculative points in the manuscript is that the results support a model in which Process S is a time-keeping mechanism that maintains a sleep quota. The data presented seem to also be entirely consistent with a model in which waking experience-dependent alterations in neuronal physiology drive deviations in firing rates which require a homeostatic regulation of this (unidentified) physiological variable. Perhaps I have missed a key differentiator between these – if so, this should be clarified for the reader. Otherwise, the speculative model should be minimized.

We agree that this was the most speculative point of our manuscript. Our initial hypothesis was indeed that local Process S expression reflects only the local need for homeostatic regulation based on the local activity history. However, while the activity-dependent models provided a generally good fit to SWA and off occupancy time courses, in both cases the fit provided by the classical state-based model was slightly superior. Since local Process S reflects global sleep-wake history a little more closely than the history of local activity, we reasoned that local Process S must reflect activity more widely, probably integrated across wider networks. Given that Process S measures depend on population synchrony, we posit that this could be the mechanism for integration. As we discuss above, one of the key open questions remaining in the field is how the constancy in daily sleep amount is maintained at the individual level. We argue that, especially in the absence of external time cues, a precise “time-keeping” mechanism is necessary, which would keep track of time spent awake and asleep. In theory, this could be an activity-dependent process, but as individual neurons and different brain regions are expected to experience widely different levels of activity, the only possibility to produce a global signal that represents sleep need across the brain is to perform integration or spatial averaging of local Processes S, each of which represents locally integrated history of activity. Although this hypothesis requires further testing, we think it may represent a tractable problem, and, to our view, represents an exciting possibility. However, following the reviewer’s recommendation we now made an effort to minimise this part to avoid any misunderstanding.

3) Much of the Discussion comes across as highly speculative.

We have reworked the Discussion and hope it is now perceived as less speculative.
